# Uptake of breast cancer screening methods: perspectives of members of staff of Federal Medical Centre, Abeokuta

**DOI:** 10.3332/ecancer.2025.1930

**Published:** 2025-06-24

**Authors:** Jessica O Esangbedo, Rachael O Oduyemi, Damilare Aduroja, Yetunde O Tola, Olajumoke Dele-Alonge, Emmanuel O Adesuyi, Michael O Ajiboye, Oluwadamilare Akingbade

**Affiliations:** 1 Faculty of Nursing Sciences, Chrisland University, Abeokuta 110104, Ogun State, Nigeria; 2 Institute of Nursing Research, Osogbo 232111, Osun State, Nigeria; 3 School of Public Health Sciences, University of Waterloo, Waterloo, ON N2L 3G1, Canada; 4 School of Nursing and Midwifery, Birmingham City University, Birmingham B15 3TN, UK; 5 Institute of Child Health College of Medicine, University of Ibadan, Ibadan 200001, Nigeria; 6 Faculty of Nursing, College of Health Sciences, University of Alberta, Edmonton T6G 1C9, Canada

**Keywords:** breast cancer, screening methods, knowledge, utilisation

## Abstract

**Introduction:**

Breast cancer (BC) was ranked the most common among the top ten malignancies in 2022, evidenced by high incidence and rates rapid mortality and morbidity rates in Nigeria. BC screening method (BCSM) helps to discover BC early, gives more treatment options and raises cancer survival rates. Little is known about the utilisation of BCSM in this community, which prompted this study.

**Objective:**

This study was conducted among the staff of the Federal Medical Center, Abeokuta, and it assessed their knowledge, attitudes and use of BCSM.

**Methods:**

This study selected 270 staff members using a descriptive cross-sectional method and a convenience sampling technique. Data were analysed using the Statistical Package for Social Sciences version 25.0. Hypotheses were tested using chi-square, multiple linear regression and Pearson correlation coefficient at a 0.05 level of significance.

**Results:**

The study’s results showed a high BCSM knowledge level of 71.9% but a low utilisation level of 57.8%; however, there was a positive attitude towards utilisation. Additionally, there was a significant relationship between staff members’ gender, age, educational qualifications, department and both their knowledge and utilisation of BCSM (p < 0.05). The Pearson correlation revealed a positive trend between knowledge and utilisation.

**Conclusion:**

BCSM offers an opportunity for early detection, diagnosis and disease prevention of BC; it also serves as an avenue to inform and enlighten people on important health issues, including health promotion activities and screening as they pertain to BC. More BC awareness programs are advocated to educate people on the importance of BC Screening to enhance early detection and treatment.

## Background

Breast cancer (BC) is a type of cancer that affects the breast tissues. It contributes to one of the most common malignancies that causes death and cancer-related morbidity in women. BC accounts for 25.3% of all new cases of cancer incidence, as it also contributes to 20.5% of all cancer-related deaths [1]. Globally, as BC is the most common disease among women, it now accounts for 25% of all new cancer cases [2]. It has been found that within the productive age of women, which is found between 25 and 50 years, BC has a more terrible effect on them during this period of their lives in which late presentation, as well as other causes that happen in most of the developing African countries, especially in Nigeria, result to the poor survival rate of this disease [3]. This, in effect, adds to the median survival of 7 months (mean 8.7 months), which is far lower than what is obtained in most developed countries in recent times [3]. Additionally, the prevalence of BC in West Africa is lower than that of North America, but the mortality rate of the disease is 50% higher, as 80% of Nigerian women with the disease show a late presentation at stages 3 and 4 of the disease [4].

As important as the knowledge of BC and BC screening methods (BCSMs) is in facilitating and encouraging screening behaviour, in sub-Saharan Africa, it has been found that the deficient knowledge of this disease has led to the lack of access to quality healthcare and late-stage diagnosis of the disease which forms a significant barrier to the effective management of BC in the region [5]. Similarly, the prevalence of a subtype of BC disease, Triple-Negative BC among black women which is characterised by the lack of three major receptors: Estrogen recepto, progesterone receptor and human epidermal growth factor receptors 2 which are usually found in other types of BC, which does not respond to the use of hormonal treatments or specific medicines targeted at these receptors according to experts is found in 1 in 5 of them [6].

Knowledge of BC and BCSM, such as breast self-examination (BSE), clinical breast examination and Mammogram, are crucial in facilitating and encouraging screening behaviour. Patient delay in seeking medical care is a significant barrier to effective BC management in Africa [5]. The major attributable factors to a low survival rate of BC in SSA include late-stage diagnosis and lack of access to quality healthcare due to a knowledge deficit on BCSM. According to the United States Preventive Services Task Force’s 2024 recommendations, screening mammograms should begin at age 40 and continue every 2 years until age 74 [7]. While studies have examined BC screening among various populations, little is known about the perspectives of members of staff of a tertiary hospital in Nigeria regarding this. As the staff of tertiary hospitals occupy a vantage position in influencing the health decisions of the public, understanding their perspectives regarding this will serve as a background for interventions to improve the uptake of BC screening among the general public.

This study aims:

1. To investigate the knowledge of BC and BCSM among the staff at the Federal Medical Centre (FMC), Abeokuta.
2. To assess the level of utilisation of BC screening methods among the population.
3. To identify the attitudes associated with the utilisation of BCSM among the population.

## Methods

### Design

This study used a cross-sectional descriptive research design.

### Setting

This study was conducted among the members of staff of FMC, Abeokuta, founded in 1983 as a tertiary healthcare institution located in Ogun State, Southwestern Nigeria. This healthcare facility comprises 36 clinical departments, 361 nurses, 166 support staff and health attendants. Also, it has 158 consultants, resident doctors, medical officers and physicians, totaling 655 healthcare workers.

### Sampling technique and sample size calculation

This research utilised a convenience sampling method. The sample size for this study was determined using the Cochran sample size formula [8], and an attrition rate of 10% to give a total of 270 members of staff.

### Inclusion and exclusion criteria

Inclusion criteria include nurses, doctors and support staff working in the clinical departments alongside healthcare workers in the Community Medicine and Primary Care at PHC. Exclusion criteria include non-health workers within the hospital, staff members off duty and on leave and unwilling and non-consenting staff members. By excluding non-health personnel, confounding variables associated with barriers in the general population are avoided and a targeted evaluation of screening uptake among medical professionals is ensured. This method makes it possible to provide more detailed, institution-specific suggestions for enhancing screening among individuals who are supposed to set an example.

### Instrument for data collection

A researcher-designed questionnaire was used to elicit data from the sampled respondents. The questionnaires were suited for the setting. The questionnaire contains 37 items in four sections: A, B, C and D.

- Section A consists of questions assessing the sociodemographic data of the respondents with ten (10) items.
- Section B consists of 13 items used to assess the staff members’ knowledge and understanding of BC, symptoms, risk factors and BCSM. This section has 13 knowledge questions with a Yes/No/Not sure structure. Yes, received a score of 1, and no/not sure received a score of 0. Scores were summed and those with a cumulation of 11–13 were considered to have a high level of knowledge, and 0–10 were considered to have a low level of knowledge.
- Section C consists of six items used to assess the participants’ utilisation of BCSM. This section has six questions presented with a Yes/No/Not sure structure. Yes, received a score of 1, and no/not sure received a score of 0. Scores were summed and those with a cumulation of 5–6 were considered to have a high level of utilisation, while 1–4 were considered to have a low level of utilisation.
- Section D consists of questions that evaluate respondents’ attitudes towards using BCSM. This section consists of 10 questions that assess potential influences on staff members’ knowledge and utilisation of BCSM. The options for these ten questions are strongly disagree, agree, neutral, agree and strongly agree. Strongly agreeing resulted in a score of ‘5’ and a positive attitude towards BCSM utilisation on positively framed questions, whereas strongly disagreeing resulted in a score of ‘1’. For negatively framed questions, strongly disagreeing resulted in a score of ‘5’ and a negative attitude towards BCSM utilisation, whereas strongly agreeing resulted in a score of ‘1’.

### Validity of the instrument

The questionnaire was subjected to face and content validity. Experts in nursing and education research, measurement and assessment got copies of the questionnaire. Before the instruments were later used, their suggestions were integrated into the final version. Using the literature review as a guide in creating the questionnaire and aligning the instrument’s items with its predetermined objectives, the face validity of the instrument was established. Afterward, the instrument was presented to the supervisor for review and potential corrections.

### Reliability of the instrument

The structured questionnaire was pre-tested among staff members from the institution who were not part of the study. The data collected were computed and analysed; thus, the reliability coefficient (Cronbach’s alpha) was computed and ambiguous items were removed before the subsequent administration. A Cronbach’s Alpha above 0.70 indicates the instrument’s reliability.

### Data analysis

The completion of the questionnaires was reviewed, and the data were collected and coded. The analysis was conducted using the Statistical Package for Social Sciences version 24. Pearson correlation coefficient, multiple regression and chi-square were used to establish the significance of the relationship between variables and results, while descriptive statistics analysis, such as percentage representations, figures, frequency tables, the mean and standard deviation, were used to present and summarise the results.

### Ethical consideration

Prior to the study, participants’ full consent was sought; neither misrepresentation of the study’s aims and objectives nor any other sort of exaggeration was used. A sufficient level of confidentiality of the research data was guaranteed, together with respect for and protection of the participants’ dignity. The participants’ anonymity was also guaranteed. Participants did not experience any kind of damage. This research proposal was submitted to the ethics committee of Federal Medical Centre, Abeokuta (FMCA), with approval number FMCA/470/HREC/01/2023/23.

## Results

A total of 270 questionnaires were distributed and retrieved. This gave a response rate of 100%. The sociodemographic characteristics of the respondents included their gender, age, religion, highest education qualification, department, location of residence, marital status, number of children, menopausal status and family history of cancer. Table 1 shows the sociodemographic characteristics of 270 sampled respondents. The sample of members of staff was mostly 205 (75.9%) female, 20–29 years 74 (27.4%), Christians 201 (74.4%), University graduates 146 (54.1%), nurses 102 (37.8%), urban residents 231 (85.6%), married 137 (50.7%), nulliparous 108 (40.0%) and premenopausal 170 (63.0%), with no family history of cancer 183 (67.8%).

### Level of knowledge and utilisation of BCSM of among participants

Table 2 shows the level of knowledge and utilisation of BCSM among the participants. The maximum knowledge score obtained was 13 (53%), while the maximum utilisation score obtained was 6 (20.7). Based on the scale, slightly less than three quarters, 194 (71.9%) of the participants had a high knowledge score of above 10 and just a little over one quarter, 76 (28.1%); however, over half, 156 (57.8%), had a low utilisation score of 4 and below.

### Attitudes toward utilisation of BCSM

Table 3 shows that the majority of participants agree that it not being shameful to have and suffer from BC and that treatment for BC is a long process. Similarly, the majority agreed that the treatment for BC is helpful to all patients, not embarrassing and that the woman can have a normal life after treatment of BC. Additionally, the majority strongly agreed to going to a doctor as soon as they feel a mass in their breast, being confident in doing a BSE, doing a BSE regularly if they know how and participating in future BC prevention programmes, as the mean value for the statements was above 4.20. The majority of the population reported disagreeing with needing someone to inform them on how to do BSE, as is seen in the mean of 2.6.

## Hypothesis testing

### Association between knowledge and utilisation of BCSM

**H1:** There is no significant association between the knowledge of BC screening methods and their utilisation.

A Pearson correlation was used to examine the association between level of knowledge and utilisation, which revealed a low positive and statistically significant result (*r* = 0.40, *p* < 0.001). This suggests that an increase in the knowledge level would lead to a higher utilisation level, as shown in Table 4.

### Relationship between the sociodemographic variables and knowledge of BC and BCSM

The study investigates the relationship between sociodemographic variables and the knowledge level of BC and BCSM. The following hypothesis was proposed:

H2 There is no significant relationship between the sociodemographic variables of respondents and the knowledge of BC and BCSM.

Chi-square statistics were used to examine the relationship between the sociodemographic variables of respondents and the knowledge of BCSM. The results of the chi-square test of Association are summarised in Table 5.

From Table 5, the respondents’ gender (*x^2^* = 5.946, *p* = 0.015), age (*x^2^* = 12.259, *p =* 0.016), highest education qualification (*x^2^* = 22.695, *p* < 0.001), department (*x^2^* = 79.463, *p* < 0.001), marital status (*x^2^* = 12.866, *p* = 0.012), menopausal status (*x^2^* = 6.348, *p* = 042) and family history of cancer (*x^2^* = 13.078, *p* < 0.001) were significantly associated with their utilisation of BCSM at *p* < 0.050. However, no significant relationship was found between the respondents’ religion, location of residence, number of children and knowledge of BC and BCSM.

### Relationship between the sociodemographic variables and utilisation of BCSM

The study seeks to investigate the relationship between sociodemographic variables and the utilisation level of BCSM. The following hypothesis was proposed:

H3. There is no significant relationship between the sociodemographic variables of respondents and the utilisation of BCSM.

A multiple linear regression analysis was conducted to examine how well sociodemographic variables can predict the utilisation level of BCSM.

The independent variables significantly predict the utilisation of BCSM, R^2^ = 0.524, F (10.259) = 28.463, *p* < 0.001, which indicates that the sociodemographic variables under study significantly impact the utilisation of BCSM. Moreover, R^2^ = 0.524 depicts that the model explains 52.4% of the variance in utilisation of BCSM, meaning a 52.4% change in utilisation of BCSM can be accounted for by sociodemographic variables.

Additionally, coefficients were further assessed to ascertain the influence of each factor on the criterion variable (utilisation of BCSM). The summary of the findings is shown in Table 6.

From Table 6, the results revealed that gender (B = 2.132, *t* = 5.632, *p* < 0.001), age (B = 0.369, *t* = 3.035, *p* = 0.003), highest education qualification (B = 0.596, *t* = 4.536, *p* < 0.001), department (B = 0.571, *t* = 5.770, *p* < 0.001) and family history of cancer (B = 0.440, *t* = 2.341, *p* = 0.020) have a significant and positive impact on utilisation of BCSM. Hence, they were supported.

However, it showed that religion (B = −0.0811, *t* = −0.400, *p* = 0.690), location of residence (B = −0.300, *t* = −1.188, *p* = 0.236), marital status (B = −0.192, *t* = −1.699, *p* = 0.091), number of children (B = −0.003, *t* = −0.025, *p* = 0.980) and menopausal status (B = 0.200, *t* = 0.691, *p* = 0.490), do not have a significant and positive impact on utilisation of BCSM. Hence, they were not supported.

## Discussion

This study assessed the knowledge and utilisation of BCSM among FMCA staff members in Ogun state. The study revealed that out of the 270 respondents, almost three-quarters had a high level of knowledge of BC and BCSM (71.9%). Similarly, over a quarter of the respondents had low knowledge of BC and BCSM (28.1%). These results agree with a study conducted by Al Mousa *et al* [9], which revealed that most participants were aware of the severity of BC and 97.7% had an intermediate to good or excellent knowledge and understanding of BC.

Furthermore, this study revealed that out of the 270 respondents, over half had a low utilisation of BCSM (57.8%), while less than half had a high utilisation of BCSM (42.2%). Despite the high level of knowledge of BCSM and its benefits, most of the population had a low level of utilisation, which may be due to the line of work, being a clinical staff and not having the luxury of time between work hours for screening utilisation. This is in line with the results, which found that only 9% of individuals regularly and monthly engage in breast self-examination; the research further explained that it was because the participants are highly occupied with their jobs, which caused screening behaviour to be insignificant [10]. Similarly, in a study conducted in Ogun State, it was reported that while staff at private tertiary institutions had good knowledge of breast self-examination, their practice was low [11]. Based on the preceding, interventions are needed to improve the utilisation of BCSM. As Zhang *et al* [12] and Maitanmi *et al* [13] highlighted the role of educational interventions in improving screening service uptake, we hereby recommend such interventions. Similarly, as Akingbade *et al* [14] and Akingbade *et al* [15] have reported high usage of mobile phones in Nigeria, we recommend the design of mobile educational interventions, which are educationally inspired technology that allows learners to learn at their own pace and convenience through mobile technologies like mobile phones. Even as Adesuyi *et al* [16] reported proficiency of nurses in Nigeria in developing digital interventions and the feasibility and acceptability of such interventions in Nigeria have been demonstrated through a pilot randomised controlled trial, nurses can play a huge role in developing such interventions [17].

The results showed that the participants had positive attitudes towards the utilisation of BCSM. The majority of the respondents were ready to go to a doctor as soon as they felt a mass in their breast and were confident in being able to perform a BSE at home by themselves if they had the proper knowledge about the procedure. This is similar to a study by Gangane and Sebastian [18], which found that over 90% of participants were ready to visit a doctor as soon as they felt a mass in their breasts, which is one of the most common symptoms of BC and that 80% of them were confident of being able to perform breast self-examination at home if they are trained to do it. Also, the findings of this current study revealed that the majority of the respondents felt they do not need someone to inform them on how to do a BSE; this is contrary to a study [18], which found that the participants needed additional education and training. The disparity may be because the sample population of the current setting all work in the clinical department at a federal hospital.

According to the findings of this study, there is a significant association between the knowledge of BC screening methods and their utilisation. This is in line with a survey to assess female healthcare workers’ knowledge, attitudes and practices regarding BC Screening (BCS), which revealed that the knowledge of healthcare professionals increased the frequency of BCS in any given population [19]. Similarly, a study by Mohan *et al* [20] showed that the percentage of participants who knew about and had ever used a BCSM was high, although regular use was low. Al Mousa *et al* [9] conducted a study that contradicts this research’s inference; the study sample had a low utilisation of BCSM while having an intermediate degree of knowledge about BCSM.

Results from this study show that a higher knowledge of BCSM was significantly related to gender, age, level of education, department and marital status, while location of residence, number of children and religion have no significant relationship with knowledge of BCSM. This is in contrast to a study by Gangane and Sebastian [18] to examine the knowledge, attitude and practices of BC among women, where a higher knowledge of BC was significantly associated with religion. However, the same study is in line with the findings of this research, which state that the location of residence has no significant relationship with knowledge of BC and BCSM. It also revealed that age and level of education are significantly associated with knowledge of BC and BCSM, alongside a study by Al Mousa *et al* [9], which found the level of education to be the primary factor affecting the knowledge of BC and BCSM.

According to the findings of this study, there is a statistically significant relationship between the utilisation of BCSM and gender, age, high level of education, department and family history of cancer, while location of residence, number of children and religion have no significant relationship with the utilisation of BCSM. This is contrary to findings by Mohan *et al* [20] and Wu *et al* [21], which both revealed a significant relationship between the residential area and utilisation of BCSM and found that participants living in rural areas were less likely to utilise BCSM in comparison with those living in urban areas. Level of education also invalidates a study by Bashirian *et al* [10], which showed that a BCSM was higher in those with lower levels of education; the study explained it is because the highly educated participants did not believe that the BCSM was useful. Also, it was indicated in the study by Bashirian *et al* [10] that participants with a family history of breast disease have a higher chance of utilising BCSM.

A high utilisation of BCSM increases health promotion and disease prevention. With increasing rates of BC morbidity all across Nigeria and the world at large, BCS is a vital component for early detection. It provides an opportunity for a timely diagnosis of breast disorders, leading to a better prognosis if diagnosed. Hence, it is crucial for healthcare workers to be continuously involved in educating people about the disease process, risk and predisposing factors, aetiology, its signs and symptoms and prevention and treatment strategies.

### Implications for practice and recommendations

1. The hospital administration can make screening more convenient and accessible for staff members by integrating routine BC screening into wellness initiatives. To increase accessibility and affordability, policymakers should encourage and fund workplace screening programs in medical institutions. Offsetting screening expenses and increasing access to screening services for healthcare professionals and the general public can be achieved through cooperation between public and commercial organisations.
2. The hospital can minimise logistical obstacles and promote staff engagement by implementing recurring on-site BC screening programs. Early detection rates can be raised by requiring BC screening as part of hospital employees’ yearly physical examinations.
3. The establishment of a ‘Caring for the Carers’ Health Check Day, where all hospital employees receive a routine physical examination, including a BC screening, is recommended. This program can ensure that healthcare professionals, who frequently spend their time attending to patients, also obtain the preventive treatment they require. Establishing a specific day every year or every 2 years for staff screenings will remove the justification of time constraints and promote broad involvement. By requiring involvement and offering rewards for compliance, the hospital administration can further encourage this endeavour.
4. The results can be utilised to create focused hospital-wide awareness efforts that dispel myths and obstacles to staff adoption of BC screening. It is expected that a high level of health promotion and disease is guaranteed if there is a rapid adoption of BCSM.

## Limitations

This study had its limitations. First, the respondents were only from the Federal Medical Centre Abeokuta clinical department, and this finding might not be generalisable to staff in non-clinical departments. Also, only one institution was used, which limits generalisation. Finally, the study’s findings were based on self-report and could be subject to bias.

Since the study only covered knowledge and utilisation of BC and BCSM, further research can extensively investigate the association between these variables and attitudes toward the utilisation of BCSM. Also, further studies to explore the effect of socio-cultural beliefs, myths and misconceptions surrounding BC and BCSM on knowledge and utilisation of BC and BCSM are hereby recommended. Qualitative studies to explore the participants’ experiences regarding the utilisation of BCSM are also recommended. Further research can study populations with a larger sample size.

## Conclusion

This study reports the knowledge and utilisation of BC screening methods among staff members in the Federal Medical Centre, Abeokuta Ogun state. As shown in this current study, BCSM offers an opportunity for early detection, diagnosis and disease prevention of BC; it also serves as an avenue to inform and enlighten people on important health issues, including health promotion activities and screening as it pertains to BC. It is hereby recommended that a carefully planned qualitative study could offer a better understanding of how the health system can increase breast awareness and support people’s engagement in cancer control and prevention initiatives. Also, more BC awareness programmes should be created by health schemes, such as the National Health Insurance Authority and Ogun State Health Insurance Agency, for all individuals, irrespective of their gender or educational attainment, to educate people on the importance of BCS for health promotion, disease prevention and early detection. To reduce the prevalence of the disease rising incidence, updated policy guidelines for raising awareness of the condition must be created.

## Conflicts of interest

The authors declare that they have no conflicts of interest.

## Funding

No external source of funding was received for this study.

## Informed consent

Participants’ consent was obtained via a consent form.

## Author contributions

Jessica O Esangbedo: primary author, co-conceived the study, determined the research design, data collection and investigation, performed data analysis and interpreted data and drafted the manuscript. Rachael O Oduyemi: supervised and co-conceived the study and participated in its design. Damilare Aduroja: drafted and revised the manuscript. Yetunde O Tola, Olajumoke Dele-Alonge, Emmanuel O Adesuyi, Michael O Ajiboye contributed to the manuscript’s text and content, including revisions and edits. Oluwadamilare Akingbade: supervised and provided revisions to the scientific content of the manuscript, offered grammatical revisions to the manuscript, participated in designing the study and participated in formal analysis and data interpretation.

All authors approve of the manuscript’s content and agree to be held accountable for the work.

## Figures and Tables

**Table 1.** Frequency distribution for sociodemographic characteristics.

Sociodemographic variables		Frequency (*N*)	Percent (%)
Gender	Female	205	75.9
	Male	65	24.1
Age	20–29	74	27.4
	30–39	81	30.0
	40–49	83	30.7
	50–59	28	10.4
	60–69	4	1.5
Religion	Christian	201	74.4
	Muslim	69	25.6
Highest education qualification	No formal education	–	0
	Primary	–	0
	Secondary	7	2.6
	Post-secondary	21	7.8
	University	146	54.1
	Post-graduate	96	35.6
Department	Nursing	102	37.8
	Medicine	59	21.9
	Support staff	109	40.4
Location of residence	Urban	231	85.6
	Rural	39	14.4
Marital status	Unmarried	102	37.8
	Married	137	50.7
	Separated	11	4.1
	Divorced	12	4.4
	Widowed	8	3.0
Number of children	0	108	40.0
	1–2	101	37.4
	3–4	48	17.8
	>5	13	4.8
Menopausal status (if female)	Pre	170	63.0
	Post	35	13.0
	Not female	65	24.1
	No	183	67.8

**Table 2.** Frequency distribution for level of knowledge and utilisation.

	Level	Scores	Frequency (*N*)	Percent (%)
Knowledge	Low	0–10	76	28.1
	High	11–13	194	71.9
Total			270	100.0
Utilisation	Low	0–4	156	57.8
	High	5–6	114	42.2
Total			270	100.0

**Table 3.** Respondents’ attitude towards utilisation of BCSM.

	Frequency (%)						Std. deviation
Statements	SA	A	*N*	D	SD	Mean	
It is not shameful to have and suffer from BC	131 (48.5%)	86 (31.9%)	23 (8.5%)	11 (4.1%)	19 (7.0%)	4.11	1.166
Treatment for BC is a long process.	107 (39.6%)	119 (44.1%)	31 (11.5%)	6 (2.2%)	7 (2.6%)	4.16	0.901
Treatment for BC is helpful in all patients and not only in young patients.	135 (50.0%)	107 (39.6%)	19 (7.0%)	9 (3.3%)	–	4.36	0.758
Treatment for BC is not embarrassing.	130 (48.1%)	105 (38.9%)	19 (7.0%)	16 (5.9%)	–	4.29	0.840
A woman treated for BC can have a normal life.	124 (45.9%)	105 (38.9%)	23 (8.5%)	15 (5.6%)	3 (1.1%)	4.23	0.904
I would go to a doctor as soon as I feel a mass in my breast.	129 (47.8%)	103 (38.1%)	31 (11.5%)	5 (11.5%)	2 (.7%)	4.30	0.802
I am confident that I can do BSE at home by myself.	165 (61.1%)	59 (21.9%)	31 (11.5%)	8 (3.0%)	7 (2.6%)	4.36	0.976
I need someone to inform me how to do BSE	20 (7.4%)	51 (18.9%)	46 (17.0%)	106 (39.3%)	47 (17.4%)	2.60	1.190
I will do BSE regularly if I know how to do it.	133 (49.3%)	97 (35.9%)	31 (11.5%)	8 (3.0%)	1 (.4%)	4.31	0.817
I would participate in future BC prevention programmes.	166 (61.5%)	85 (31.5%)	14 (5.2%)	1 (.4%)	4 (1.5%)	4.51	0.745

Note SA = strongly agree, A = agree, N = neutral, D = disagree, SD = strongly disagree

**Table 4.** Correlations between variables of interest.

	Knowledge level	Utilisation level
**Knowledge level**	.	0.40**
**Utilisation level**	0.40**	.

Note ** Correlation is significant at the 0.01 level (2-tailed)

**Table 5.** Chi-square analysis results of the relationship between sociodemographic variables and Knowledge.

Sociodemographic variables		Level of knowledge		*x* ^2^	df	*p*
		Low	High			
Gender	Female	50 (24.4%)	155 (75.6%)	5.946	1	0.015*
	Male	26 (40.0%)	39 (60.0%)			
Age	20–29	31 (41.9%)	43 (58.1%)	12.259	4	0.016*
	30–39	16 (19.8%)	65 (80.2%)			
	40–49	18 (21.7%)	65 (78.3%)			
	50–59	10 (35.7%)	18 (64.3%)			
	60–69	1 (25.0%)	3 (75.0%)			
Religion	Christian	62 (30.8%)	139 (69.2%)	2.830	1	0.093
	Muslim	14 (20.3%)	55 (79.7%)			
Highest Education Qualification	No formal education	–	–	22.695	3	0.000*
	Primary	–	–			
	Secondary	7 (100.0%)	0 (0.0%)			
	Post-secondary	9 (42.9%)	12 (57.1%)			
	University	40 (27.4%)	106 (72.6%)			
	Post-graduate	20 (20.8%)	76 (79.2%)			
Department	Nursing	8 (7.8%)	94 (92.2%)	79.463	2	0.000*
	Medicine	5 (8.5%)	54 (91.5%)			
	Support staff	63 (57.8%)	46 (42.2%)			
Location of residence	Urban	65 (28.1%)	166 (71.9%)	0.000	1	0.993
	Rural	11 (28.2%)	28 (71.8%)			
Marital status	Unmarried	32 (31.4%)	70 (68.6%)	12.866	4	0.012*
	Married	32 (23.4%)	105 (76.6%)			
	Separated	6 (54.5%)	5 (45.5%)			
	Divorced	1 (8.3%)	11 (91.7%)			
	Widowed	5 (62.5%)	3 (37.5%)			
Number of children	0	35 (32.4%)	73 (67.6%)	4.663	3	0.198
	1–2	24 (23.8%)	77 (76.2%)			
	3–4	11 (22.9%)	37 (77.1%)			
	>5	6 (46.2%)	7 (53.8%)			
Menopausal status (if female)	Pre	43 (25.3%)	127 (74.7%)	6.348	2	0.042*
	Post	7 (20.0%)	28 (80.0%)			
	Not female	26 (40.0%)	39 (60.0%)			
Family History of cancer	Yes	12 (13.8%)	75 (86.2%)	13.078	1	0.000*
	No	64 (35.0%)	119 (65.0%)			

*x*^2^ = Pearson chi square value, df = degree of freedom, P = Probability value, *: significant at *p* < 0.050

**Table 6.** Multiple linear regression of the relationship between sociodemographic variables and Utilisation.

Sociodemographic variables	B	t	*p*-value	Results
Gender	2.132	5.632	0.000*	Supported
Age	0.369	3.035	0.003*	Supported
Religion	–0.0811	–0.400	0.690	Not supported
Highest education qualification	0.596	4.536	0.000*	Supported
Department	0.571	5.770	0.000*	Supported
Location of residence	–0.300	–1.188	0.236	Not supported
Marital status	–0.192	–1.699	0.091	Not supported
Number of children	–0.003	–0.025	0.980	Not supported
Menopausal status (if female)	0.200	0.691	0.490	Not supported
Family history of cancer	0.440	2.341	0.020*	Supported
R^2^	0.524			
F (10.259)	28.463			

Dependent Variable: Utilisation Score, *: significant at *p* < 0.050
